# Evaluation of Biodentine in Pulpotomies of Primary Teeth with Different Stages of Root Resorption Using a Novel Composite Outcome Score

**DOI:** 10.3390/ma14092179

**Published:** 2021-04-24

**Authors:** Rosa Guagnano, Federica Romano, Patrizia Defabianis

**Affiliations:** 1 Department of Surgical Sciences, C.I.R. Dental School, Section of Pediatric Dentistry, University of Turin, 10126 Turin, Italy; rosa.guagnano@unito.it; 2 Department of Surgical Sciences, C.I.R. Dental School, Section of Periodontology, University of Turin, 10126 Turin, Italy; federica.romano@unito.it

**Keywords:** biomaterials, pulpotomy, pulp vitality, primary teeth, root resorption

## Abstract

This study aimed to assess the success of pulpotomy in primary molars using Biodentine, new-developed tri-calcium, di-calcium-based silicate cement, at 6 and 12 months. The hypothesis was that stages of root resorption could influence the treatment success. A novel composite score was used based on five clinical and radiographic outcomes: soft-tissue pathology, pain to percussion, pathologic mobility, radiolucency and pathologic root resorption. Patients’ compliance and intraoperative pain experience were recorded using the Frankl scale and the Wong–Baker scale. A total of 22 primary molars, 9 in stage S (stability) and 13 in stage R (resorption) were submitted to pulpotomy using Biodentine and restored with composite resin. The success rate was 92.3% in the R group compared to 100% in the S group at both 6 and 12 months (*p* = 0.850). There was no statistically significant effect of type of molar, tooth position and type of carious lesions on the composite outcome (all *p* > 0.05). Overall, 73% of the children experienced no or mild/moderate pain and 77% had a cooperative attitude. Children younger than 7 years old experienced more pain (*p* = 0.04). Biodentine is a promising biomaterial for pulpotomy of primary teeth regardless of the stage of root resorption.

## 1. Introduction

Nowadays, dental caries in primary teeth continues to be an important oral health problem which affects a large part of the worldwide child population, with an overall prevalence of 46.2% [1]. The maintenance of deciduous teeth until exfoliation represents a crucial goal in pediatric dentistry to prevent aesthetic, phonetic and functional problems and to preserve the space for the eruption of permanent teeth [2]. 

Dental decay in primary teeth can range from enamel demineralization to partial loss of tooth structure, and up to complete crown destruction. In cases of deep carious lesions close to the pulp, without evidence of pulp degeneration, the treatment options are indirect pulp capping, direct pulp capping or pulpotomy [3]. Pulpotomy is indicated when the vital pulp is accidentally exposed during cavity preparation or a result of tooth fracture. It involves the removal of coronal pulp and the application of a biocompatible material on the remaining canal pulp in order to preserve its vitality until tooth exfoliation [4]. Contraindications are prolonged and excessive pulp bleeding after pulp amputation or evidence of irreversible pulpitis and pulp necrosis requiring endodontic treatment. 

Among different materials used for pulpotomy in primary teeth [5], formocresol, a fixative agent, was regarded for a long time as the gold standard, but its use was prohibited in 2004 due to teratogen effects [6]. Calcium hydroxide is not a suitable agent. Although it stimulates dentin bridge formation and has antibacterial activity, it can cause internal root resorption due to its alkalinity [7,8]. Analogously, ferric sulphate, known for its hemostatic effect, can enhance root resorption due to the non-biocompatibility of the zinc oxide layer covering the pulp [9]. 

Recently, mineral trioxide aggregate (MTA), containing tricalcium silicate, tricalcium aluminate, and calcium oxide has been introduced in endodontic treatment thanks to its high sealing ability, biocompatibility and bioinductive potential in pulp repair and dentin barrier formation [10,11,12]. The American Academy of Pediatric Dentistry has recommended it as the material of choice for pulpotomy of primary teeth [13]. Despite their high success rates, MTA-based cements have some drawbacks, including long setting times, difficulties in clinical manipulation, low share bond values, weak connection to restorative materials and high costs [14,15,16,17,18,19,20,21]. Another concern is their high discoloration potential due to the reduction or oxidation of bismuth oxide, used as a radiopacifier, when in contact with dentin collagen and sodium hypochlorite [4]. 

In this context, a recent Cochrane review highlighted the need for more clinical studies on the use of tri-calcium, di-calcium-based cements as alternative to MTA [22]. Biodentine is a powder-liquid mixture containing tri-calcium silicate, di-calcium silicate (main and second core material), calcium carbonate, zirconium oxide and iron oxide. It has antibacterial, bioinductive and biocompatible properties similar to MTA, with the advantages of simple manipulability, superior color stability due to the incorporation of zirconium oxide instead of bismuth oxide, a setting time of 12 min and high compressive and flexural strengths [23,24,25]. While promising results are emerging, little is known about its use on primary teeth in different evolutionary stages. According to some authors, physiologic root resorption greater than 75% may represent a contraindication to pulpotomy due to the elicited inflammatory reaction [26]. Other studies recommend this treatment only in cases of expected physiological exfoliation of the affected tooth in the next one or two years [27]. This is a clinically relevant topic because primary teeth undergo a rapid progression of tooth decay due to their anatomic features, and pulpotomy should preserve their vitality until their natural exfoliation, avoiding pulpectomy procedures [5].

Indeed, primary teeth have the unique feature of going through three physiologic stages that influence their reaction to different aggressions. Stage M (maturation) is the period of root formation in which the maturing pulp has dentinogenetic and repair potential. Stage S (stability) corresponds to the maturity period, while stage R (resorption) consists of the physiological root resorption process, until primary tooth substitution by the permanent successor [28,29]. 

In view of the paucity of data available in the literature, the primary aim of this prospective clinical trial was to evaluate whether the evolutionary stages of root resorption could influence the success rate of pulpotomy in primary molars using Biodentine. We introduced a novel composite score made of five clinical and radiological outcomes as proposed by Smail-Faugeron et al. [30] that may be applied in future studies on the same topic. The second aim was to evaluate whether the age of the patients could influence the treatment outcome, focusing on compliance and pain experience using the Frankl and Wong–Baker scales. 

## 2. Materials and Methods 

### 2.1. Study Design

The present study was conducted on a group of 22 healthy children (13 males and 9 females) with a mean age of 7.3 ± 1.7 years presenting 22 deciduous molars (15 maxillary and 7 mandibular, 8 first and 14 s molars) requiring pulpotomy because of deep carious lesions. Subjects participating in the study were serially recruited among patients seeking treatment at the Section of Pediatric Dentistry, C.I.R. Dental School, Department of Surgical Sciences, University of Turin (Italy) between October 2019 and January 2020. The protocol was approved by the Institutional Ethics Committee of the “AOU Città della Salute e della Scienza”, Turin, Italy and the study was conducted according to the Helsinki Declaration of 1975 (revised in 2002). Informed consent was obtained from the parents/guardians of each child before entering the study. 

The following inclusion criteria were considered: (i) age between 5 and 11 years; (ii) having at least one primary molar with a deep carious lesion approximating the pulp; (iii) no spontaneous symptoms or presence of pain stimulated by thermal or mechanical stimuli. Exclusion criteria were: (i) any concurrent medical condition; (ii) traumatic pulp exposure; (iii) previously restored or treated teeth; (iv) signs or symptoms of irreversible pulpitis (e.g., spontaneous pain) or pulp necrosis (e.g., swelling or fistula); (v) external or internal pathologic root resorption or peri-radicular radiolucency as detected on radiographs; (vi) orthodontic treatment. 

All patients underwent an intraoral examination by a single calibrated operator for cavity diagnosis and a preoperative intraoral periapical radiograph using Kodak^®^ intraoral machine (model 2100) set at 70 Kv with 0.3 s of exposure time for maxillary molars and 0.2 s for mandibular molars. The tooth evolutionary physiological stage was diagnosed on preoperative intraoral radiographs as follows: stage M when at least one of the roots was immature; stage S when all roots were completely developed or showed a physiologic resorption less than 75% of their normal length; stage R when at least one of the roots presented a reabsorption more than 75% of its normal length. Patients were divided into 3 age groups: under 7 years of age, between 7 and 9 years, and over 10 years of age.

### 2.2. Clinical Procedures

**Figure 1:**
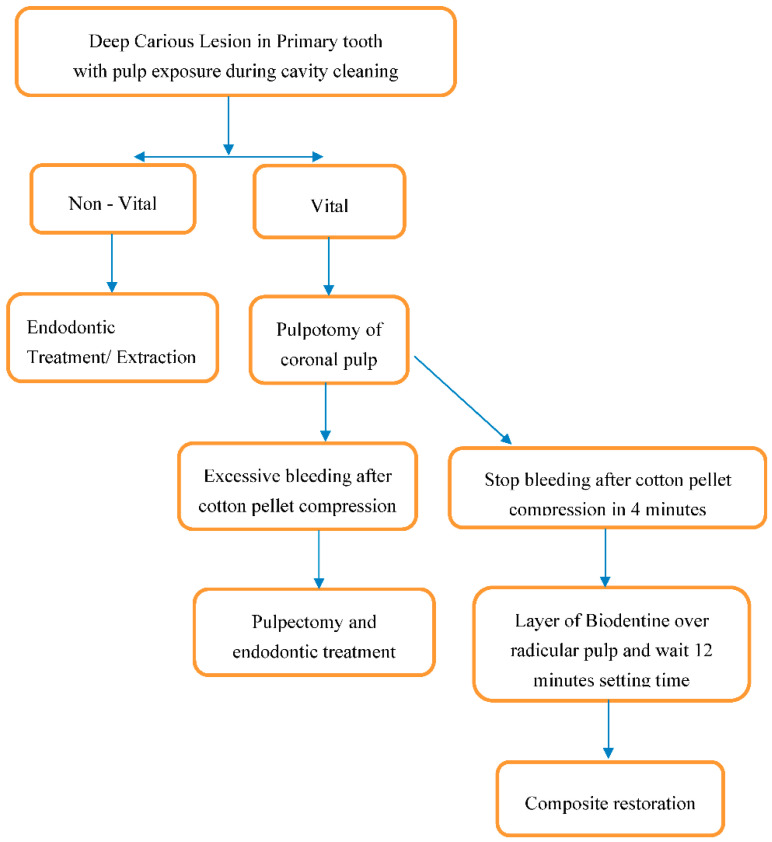
Flowchart summarizing the decision process and the clinical phases of the pulpotomy procedure.

A single specialist in pediatric dentistry performed all the pulpotomy procedures under the same conditions and using the same tools following the steps summarized in Figure 1.

After local anesthesia and isolation of the decayed primary molar under a rubber dam, all caries was removed with a cylindrical diamond bur in a high-speed handpiece under copious water spray, using a magnifying loupe (2.5×). The pulp chamber was opened with a non-active conic diamond bur and the removal of the coronal pulp tissue was performed using a carbide round bur in a low-speed handpiece. Pulp bleeding was controlled by compression with a cotton pellet soaked in sterile saline at room temperature for 5 min. Once hemostasis was obtained, a layer of Biodentine (Biodentine^TM^, Septodont, Saint Maur-des-Fossés, France) was applied over the amputated root pulp. The cement was prepared mixing 5 drops of liquid with the powder contained in the premeasured unit dose capsules for 30 s at 4200 rpm. After 12 min, the cavity was etched with 37% orthophosphoric acid for 40 s and then rinsed with water for 80 s. An acetone-based single-bottle adhesive (Prime&Bond NT, DeTrey/Dentsply, Konstanz, Germany) was applied on the dry cavity with a microbrush and light cured for 20 s. A thin layer of flowable microfilled hybrid composite resin (Gradia Direct Flow, GC, Alsip, IL, USA) was carefully placed over the pulp chamber and polymerized for 40 s. The cavity was filled with incremental apposition of 2 mm of macro-filled composite resin (Filtek, 3M Oral Care, Conway, MN, USA), after a polymerization of 40 s each time until the restoration was completed.

### 2.3. Clinical and Radiographic Outcomes

**Figure 2:**
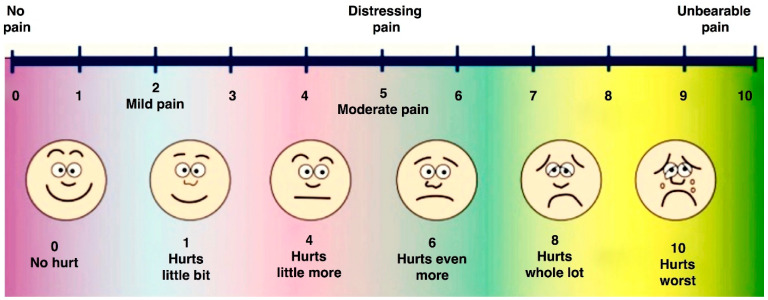
Visual analog scale according to Wong–Baker faces scale (1988).

At the completion of the pulpotomy, the child was asked to score the intensity of the intraoperative pain or discomfort according to the Wong–Baker visual analog scale (Figure 2), specifically created for pediatric patients [31]. A score from 0 to 3 is indicative of light pain, from 3 to 6 of moderate pain, and from 7 to 10 of severe pain. 

**Figure 3:**
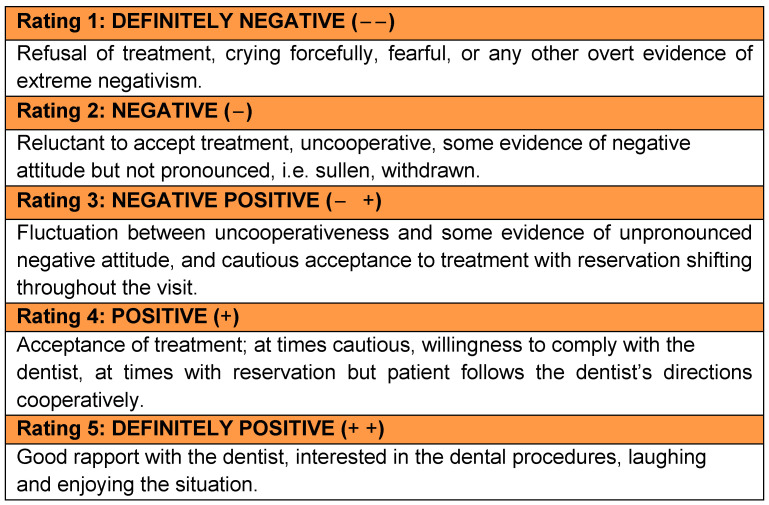
Modified Frankl scale (1975) to rate the degree of patient’s cooperation.

The operator rated the degree of the patient’s cooperation using the modified Frankl scale [32] that classifies the patient’s behavior on a 1–5 point scale where 1 represented definitely negative and 5 a definitely positive attitude as described in Figure 3. 

At 3, 6 and 12 months postoperatively, the presence/absence (1/0) of soft tissue pathology, pain to percussion and pathologic mobility were recorded. 

At 6 and 12-month follow-up visits, intraoral periapical radiographs were also taken to evaluate the presence/absence (1/0) of pathologic radiolucency and pathologic root resorption. These criteria allowed for assessing the conditions of tooth, alveolar bone and periodontium. To assess globally the success of pulpotomy at 6 and 12 months postoperatively, a composite score was created combining the 5 above-mentioned clinical and radiological outcomes [30]. The treatment was deemed as unsuccessful even if one of these criteria scored positive.

At 6 months, to assess the intra- and inter-examiner repeatability, the re-examination of 10 participants was performed on the composite score. Cohen’s Kappa statistical test resulted in excellent intra- and inter-examiner agreement (0.92 and 0.87, respectively).

### 2.4. Statistical Analysis

Statistical analyses were performed using software (SPSS Statistics for Mac, v. 25.0, IBM, Chicago, IL, USA). Data were analyzed, considering the clinical and radiographic outcomes separately at 3, 6 and 12 months and their combination in the composite score. 

The statistical significance of the differences between the groups [age, stage of root resorption, type of primary molar (first/second), tooth position (mandibular/maxillary arch), type of cavity (occlusal/interproximal) was evaluated using the Chi-Square test or Fisher exact test for qualitative variables. Distribution of quantitative variables was analyzed with the Mann–Whitney U test or Kruskal–Wallis test as appropriate. *p* values less than 0.05 were considered statistically significant for all the analyses.

## 3. Results

### 3.1. Clinical and Radiographic Outcomes

Table 1 summarizes clinical and radiographic outcomes over the study period. Deep carious lesions involved the occlusal surface in 9% (2 teeth), and the interproximal area in 91% of the cases (20 teeth). Nine primary molars were in S stage and 13 in R stage, while none were in M stage.

At 3 months, all clinical outcomes were negative, with no cases of swelling, fistula, pain or pathologic mobility.

At 6 months, 95.5% of the cases (21 primary molars) presented both clinical and radiological success. One deciduous second mandibular molar in stage R had exfoliated physiologically and the permanent successor tooth had already erupted. Only one primary maxillary molar in stage R presented fistula, pathologic mobility and pain to percussion. Radiologically, it was the only tooth showing peri-radicular radiolucency with signs of secondary infiltration of the interproximal composite restoration. This tooth was submitted to root canal treatment. 

**Figure 4:**
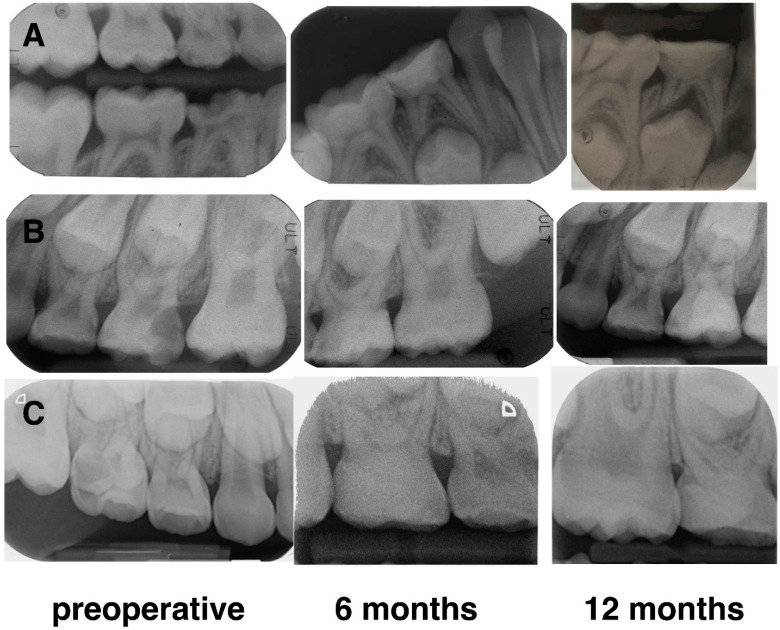
Radiographic images of successful pulpotomy of three primary molars at 6 and 12 months. Case (**A**): lower right first molar in stage S in a 6-year-old male; Case (**B**): upper left second molar in stage R in a 10-year-old female; Case (**C**): upper right second molar in stage S in a 6-year-old female.

At 12 months, 95.5% of the cases (21 primary molars) showed clinical and radiological success; two maxillary second molars in stage R were exfoliated physiologically without any clinical symptoms and the permanent teeth had already erupted. Figure 4 shows three cases of pulpotomy performed in S and R stages of root resorption.

There was no statistically significant difference between the evolutionary root resorption stage groups either for the composite outcome score at 6 and 12 months (both *p* = 0.850) or for the physiological exfoliation at the same time points (*p* = 0.850 and *p* = 0.494, respectively). The success rate was 92.3% in the R group compared to 100% in the S group at both 6 and 12 months. 

In addition, there was no statistically significant effect of type of molar, tooth position and type of carious lesions on the composite outcome (all *p* > 0.05). 

### 3.2. Patient-Related Outcomes

Table 2 describes pain perception and attitudes among the different age groups. Of the 21 children, 9 were under 7 years of age, 10 were between 7 and 9 years of age and 3 were over 10 years of age. 

According to the Wong–Baker visual analog scale, 5 patients (22.7%) did not perceive any pain and 11 (50%) reported mild/moderate pain during treatment, while only 6 patients (27.3%) experienced intense pain. Patients under 7 years of age reported higher pain scores compared to older patients; the difference was statistically significant (*p* = 0.041).

During the procedure, the operator observed an intermediate cooperation in 5 cases (22.7%), while 15 children (68.2%) were cooperative and 2 (9.1%) were absolutely cooperative. The difference in behavior in the three groups of age was statistically significant (*p* = 0.006), with the younger groups under 7 years of age and between 7 and 9 years showing a lower degree of cooperation compared to the older group.

As reported in Table 3, pain perception and attitudes were not related to the evolutionary root resorption stages. 

## 4. Discussion

Pulpotomy treatment is the preferred clinical procedure to preserve deciduous molars in cases of coronal pulp exposure caused by caries, cavity preparation or trauma, but it requires a rigorous selection of cases for a successful outcome [13]. It consists of the removal of the vital coronal pulp tissue and the isolation of the remaining radicular dental pulp using a biocompatible and antibacterial material that protects the pulp from further injury and promotes healing [13]. 

Biodentine has characteristics similar to natural dentin, has bioactive properties and elicits no sign of moderate to severe inflammatory response that may lead to irreversible changes in the pulp status [33]. It enables the stimulation of growth factors that promote differentiation of odontoblasts and dentinogenesis. A recent study reported that the reparative dentin bridge observed after direct pulp capping presented dentin tubules and chemical composition similar to primary dentin [34]. Several studies have demonstrated comparable clinical outcomes to MTA when Biodentine was used in pulpotomy of primary molars with a global success rate ranging from 94% to 100% at 12 months [33,35,36]. 

It is noteworthy that clinical and radiological parameters used to assess the efficacy of vital pulp therapies are very heterogeneous in the literature and consensus is lacking on the most relevant outcomes to be evaluated. The present study proposes a novel composite score combining five clinical and radiographic outcomes to define success/failure of pulp treatment in primary teeth that has the advantage of being synthetic and easy to use, and thus it could be useful for comparing results across future trials on the same topic. The selection of these component outcomes was based on a systematic review of pulp treatment techniques on primary molars [30]. 

Although pulpotomy has been indicated as a vital pulp treatment for deciduous teeth in the three physiological stages of root resorption, only two studies investigated its efficacy using Biodentine on stage S and R molars, respectively [37,38]. Both achieved a success rate of 100% at 12 months. To the best of our knowledge, this is the first study comparing the 12-month success rate of pulpotomy carried out by a single clinician under the same experimental conditions on primary molars in stages S and R using Biodentine. Interestingly, the treatment outcome was not influenced by the physiological root status. Radiographically, no root resorption, and no periapical radiolucencies were detected. In addition, there were no obvious changes to the pericoronal sac associated with the underlying developing permanent teeth. These findings demonstrate that, despite the low healing potential of deciduous molars in stage R, the tri-calcium silicate cement could ensure stability and pathology-free frames until the physiological exfoliation of the primary molars and the eruption of their successors. In an in vitro study, it was observed that Biodentine exhibited better cytocompatibility and bioactivity than MTA on stem cells from human exfoliated primary teeth [39].

Only two teeth (9%) in stage R exfoliated physiologically after 6 and 12 months, without any significant difference compared to molars in stage S, emphasizing that this treatment does not interfere with physiological root resorption. According to Coll et al., a tooth exfoliated at least six months following vital pulp therapy should be considered a success [40]. One failure occurred at 6 months and was related to secondary caries. 

Indeed, the global success rate was 95.4% at 12 months, slightly lower than percentages reported by Nasseh et al. [37] and Nasrallah et al. [38]. It should be taken into account that they used stainless steel crowns (SSC) for the final restoration. In the present study, restoration with composite material was preferred to the SSC or amalgam, because it was possible to perform adhesive reconstruction that provides good aesthetic results with suitable closure and lower cost. While some studies reported that SSC increase the success rate of pulpotomy, others did not find any significant differences compared to teeth treated with conservative restorations [35,41,42]. The final restoration was made during the same appointment of the pulpotomy procedure. Biodentine does not need the use of separate restoration due to its high shear bond strength to resin-based composite [43,44].

Few studies in the literature evaluated patients’ outcomes using the Frankl and Wong–Baker scales [32,45]. They reported that the majority of children were cooperative but experienced post-operative pain irrespective of the pulpotomy agent used, suggesting that placing SSC might be the major etiologic factor for the post-operative pain [46]. Consistent with these findings, most of the children submitted to pulpotomy in the present study referred no/mild pain (73%) and were cooperative (77%). It is noteworthy that there was a statistically significant difference between classes of age, with patients younger than 7 years old experiencing more intense pain (*p* = 0.04). Although the Wong–Baker faces scale does not require the ability to quantify pain because children simply match how they feel to one of the faces, preschool children have the tendency to use the extreme of the scale more often. Their self-reports are influenced by their previous painful experiences and by the perception of the consequence of their ratings [46]. 

Interestingly, patients below 9 years of age were less cooperative (*p* = 0.006). Behavior of children in the dental setting would seem to be related to age, dental history and the complexity of the dental procedure. In most cases, younger children tend to be less cooperative than older ones. More invasive procedures typically result in worse behavior outcomes and generally cooperation declines through the course of the appointment [47,48].

The present high success rate of pulpotomy with Biodentine™ can be related to proper case selection, the antiseptic standard with correct isolation under rubber dam of all the treated teeth, the high sealant capacity and mechanical properties of Biodentine, and the adequate marginal seal of the adhesive resin composite and the immediate tooth restoration. However, the rigorous selection of cases is fundamental, because not all teeth can be treated with pulpotomy procedures. According to some authors, the occurrence of intense bleeding after exposure is suggestive of a pulp tissue at a moderate to severe inflammatory stage, and therefore does not indicate pulpotomy [49]. 

Another clinically relevant advantage of Biodentine is the faster setting time that allows the operator to complete the treatment in one single visit, thus reducing the risk of bacterial contamination and the number of appointments for the children. 

The limitations of the present study are the small sample size and the convenience sampling of children among those referred to a university-based outpatient dental clinic. The short-time follow-up and the lack of a control group should also be acknowledged. However, it should be taken into account that most clinical and radiographic failures of pulpotomy treatment in primary molars occur in the first 9 months after treatment [41]. Furthermore, several studies have already demonstrated that Biodentine performs equal to MTA-based cement with comparable success rate [32,34,35]. 

The strengths of the present study include the use of a novel composite outcome that could be applied to rate the clinical and radiographic success of vital pulp therapies and the Biodentine application in different physiological root development stages of primary molars. 

## 5. Conclusions

Based on the findings of the present study, Biodentine is a suitable material for pulpotomy in primary molars irrespective of the stage of root resorption, with a success rate of 95.5% at 12 months. Pulpotomy is a painless procedure well accepted by pediatric patients, although children younger than 7 years old are less cooperative and perceived more intense intra-operative pain. This article provides insightful information on the use of new biomaterials for pulp treatment of primary teeth that can be relevant to clinical practice. Further studies with a larger sample size and a longer follow-up are needed to confirm these initial results using the same core set of composite outcomes and performing a cost-effectiveness analysis.

## Figures and Tables

**Table 1:** Clinical and radiological outcomes and composite score outcomes at 3, 6 and 12 months.

OUTCOMES	3 MONTHS			6 MONTHS			12 MONTHS		
**Clinical**	**+**	**−**	**NA**	**+**	**−**	**NA**	**+**	**−**	**NA**
Soft tissue pathology	0	22 (100%)	0	1 (4.5%)	20 (90.9%)	1 (4.5%)	1 (4.5%)	19 (86.3%)	2 (9.0%)
Pain to percussion	0	22 (100%)	0	1 (4.5%)	20 (90.9%)	1 (4.5%)	1 (4.5%)	19 (86.3%)	2 (9.0%)
Pathologic mobility	0	22 (100%)	0	1 (4.5%)	20 (90.9%)	1 (4.5%)	1 (4.5%)	19 (86.3%)	2 (9.0%)
**Radiological**	**+**	**−**	**NA**	**+**	**−**	**NA**	**+**	**−**	**NA**
Pathologic radiolucency	-	-	-	1 (4.5%)	20 (90.9%)	1 (4.5%)	1 (4.5%)	19 (86.3%)	2 (9.0%)
Pathologic root resorption	-	-	-	1 (4.5%)	20 (90.9%)	1 (4.5%)	1 (4.5%)	19 (86.3%)	2 (9.0%)
Composite score				1 (4.5%)	20 (90.9%)	1 (4.5%)	1 (4.5%)	19 (86.3%)	2 (9.0%)

+: Positive; −: Negative; NA: not applicable, defining the cases that could not be evaluated due to physiologic factors such as the eruption of successors.

**Table 2:** Pain perception and attitude [*n* (%)] using Wong–Baker and Frankl scales, respectively, among the different age groups and corresponding *p* values.

Age	Wong-Baker Scale			*p* Value	Frankl scale					*p* Value
	No	Mild/Moderate	Severe		Definitely Negative	Negative	Intermediate	Positive	Definitely Positive	
5–6 years	0	4 (36.4)	5	0.041	0	0	3 (60)	6 (40)	0	0.006
7–9 years	3 (60)	6 (54.5)	1		0	0	2 (40)	8 (53.3)	0	
10–11 years	2 (40)	1 (9.1)	0		0	0	0	1 (6.7)	2 (100)	
Total	5	11	6		0	0	5	15	2	

**Table 3:** Pain perception and attitude [*n* (%)] using Wong–Baker and Frankl scales, respectively, in the different evolutionary root resorption stages and corresponding *p* values.

Stage	Wong-Baker scale			*p* Value	Frankl Scale					*p* Value
	No	Mild/Moderate	Severe		Definitely Negative	Negative	Intermediate	Positive	Definitely Positive	
S	2 (40)	4 (36.4)	3 (50)	0.860	0	0	3 (60)	5 (33.3)	1 (50)	0.555
R	3 (60)	7 (63.6)	3 (50)		0	0	2 (40)	10 (66.4)	1 (50)	
Total	5	11	6		0	0	5	15	2	

S: stability stage; R: resorption stage.

## Data Availability

The data presented in this study are available upon request from the corresponding author.
